# Knowledge, Attitudes, and Practices Regarding Avian Influenza A (H7N9) Among Mobile Phone Users: A Survey in Zhejiang Province, China

**DOI:** 10.2196/mhealth.3394

**Published:** 2015-02-04

**Authors:** Hua Gu, Zhenggang Jiang, Bin Chen, Jueman (Mandy) Zhang, Zhengting Wang, Xinyi Wang, Jian Cai, Yongdi Chen, Dawei Zheng, Jianmin Jiang

**Affiliations:** ^1^Zhejiang Provincial Center for Disease Control and PreventionHangzhouChina; ^2^New York Institute of TechnologyDepartment of Communication ArtsOld Westbury, NYUnited States

**Keywords:** influenza A virus, subtype H7N9, knowledge, attitude, surveillance

## Abstract

**Background:**

Understanding people’s knowledge, attitudes, and practices (KAP) regarding a new infectious disease is crucial to the prevention and control of it. Human infection with avian influenza A (H7N9) was first identified on March 31, 2013 in China. Out of the total number of 134 cases confirmed from March to September 2013 in China, Zhejiang Province saw the greatest number (46 cases).

**Objective:**

This study employed a mobile Internet survey to assess KAP regarding H7N9 among mobile phone users in Zhejiang Province. This study intended to examine KAP by region and the association between sociodemographic variables and KAP.

**Methods:**

An anonymous questionnaire was designed by Zhejiang Provincial Center for Disease Control and Prevention (CDC). A cross-sectional survey was executed through a mobile Internet application platform of China Unicom in 5 regions in Zhejiang Province. Stratified and clustered sampling methods were applied and mobile phone users were invited to participate in the study voluntarily.

**Results:**

A total of 9582 eligible mobile phone users participated in the survey with a response rate of 1.92% (9582/5,000,000). A total of 9105 valid responses (95.02%) were included for statistical analysis. Generally, more than three-quarters of the participants had some basic knowledge of H7N9 and held the attitude recommended by the Zhejiang CDC toward eating cooked poultry (77.55%, 7061/9105) and visiting a hospital at the occurrence of symptoms (78.51%, 7148/9105). Approximately half of the participants worried about contracting H7N9, and took preventive practices recommended by the Zhejiang CDC. But only 14.29% (1301/9105) of participants kept eating cooked poultry as usual. Although worry about H7N9 infection did not differ by region, Hangzhou saw the largest proportion of participants with knowledge of H7N9, which was probably because Hangzhou had the greatest number of H7N9 cases. KAP varied by some sociodemographic variables. Female participants were more likely to know about symptoms of H7N9 (OR 1.32, 95% CI 1.08-1.61), to worry about contracting it (OR 1.15, 95% CI 1.04-1.27), and to report their lives being influenced by it (OR 1.27, 95% CI 1.15-1.41). They were also more likely to take the recommended precautions. Male participants and younger participants were less likely to comply with advocated protective practices.

**Conclusions:**

The results suggest that health education should be customized depending on sociodemographic variables to achieve more effective behavioral outcomes.

##  Introduction

On March 31, 2013, the Chinese government announced 3 cases of human infection with the avian influenza A (H7N9) virus in 2 provinces [1]. From March to September 2013, a total of 134 cases were identified in 12 provinces with Zhejiang Province seeing the largest number 46 cases, including 11 deaths [2]. The number of confirmed cases in Hangzhou, the capital city of Zhejiang Province, was the largest [2]. Most H7N9 patients had reported recent exposure to live poultry [1]. Clinical symptoms at the onset of the illness include a high fever (≥38°C) and a cough. As the disease progressed, patients could develop dyspnea or severe progressive pneumonia, and could rapidly develop acute respiratory distress syndrome [2-4].

Given China’s high population density, prevention and control of new infectious diseases is particularly challenging. There was limited knowledge available about H7N9 when it struck, which raised many public health concerns globally [5]. To prevent and control H7N9, the Zhejiang Province Government implemented a series of interventions, including increasing the emergency response level and closing live poultry markets. During the outbreak of H7N9, the Zhejiang Provincial Center for Disease Control and Prevention (CDC) used different means to conduct health education to encourage the public to take preventive behaviors. For example, the Zhejiang CDC published a booklet titled “Common Respiratory Infectious Diseases Booklet.” The printed booklets were given away in communities and the electronic version was uploaded to the Zhejiang CDC’s website for downloading. The Zhejiang CDC made full use of major media outlets, such as *Zhejiang Daily* and *Zhejiang Television*, and they also set up a 24-hour hotline. In addition, the Zhejiang CDC used 2 accounts, named China Health Education and Zhejiang Health Education, on Sina Weibo, a Twitter-like Chinese microblog, to disseminate information about behaviors to prevent respiratory communicable diseases.

Inaccurate information and negative attitudes toward emerging communicable diseases may lead to unnecessary concerns, rumors, social chaos, and even excessive panic, which might aggravate the epidemic [6]. Quick-spreading rumors of the outbreak of H7N9 were found in most provinces in China. Previous experience with severe acute respiratory syndrome (SARS) has demonstrated the importance of monitoring public perception of epidemic control, which may affect the public’s compliance to advocated precautionary behaviors. Elucidation of factors that may affect precautionary behaviors, such as knowledge and risk perception of the disease, may also help to prevent the spread of infection. Previous studies have demonstrated the positive correlation between perceived infectiousness and the willingness to adhere to recommended behaviors that prevent and control infection [7,8]. Therefore, it is important to assess the public’s knowledge, attitudes, and practices (KAP) regarding H7N9 [9]. Specifically, this study evaluated KAP among mobile phone users in Zhejiang Province. Findings of the current study can provide information about the effectiveness of the Zhejiang CDC’s health education efforts as well as how to design evidence-based interventions to reduce the risks of contracting H7N9 among the public.

Examining KAP in traditional ways, such as paper-and-pencil questionnaires, involves a great amount of human and material resources and can also take a long time. Considering that increasingly more people used mobile phones to access the Internet, we conducted a mobile Internet survey. According to the China Internet Network Information Center [10], Zhejiang Province had 65.22 million mobile phone users in January 2013, of which 4.81 million were mobile Internet users [11].

## Methods

### Overview

Zhejiang Provincial CDC designed and conducted the cross-sectional and closed survey among mobile phone users in Zhejiang Province regarding H7N9 during the outbreak of this new avian influenza. We described the survey and reported results according to “Checklist for Reporting Results of Internet E-Surveys (CHERRIES)” [12].

### Study Site

We performed this study in 5 regions in Zhejiang Province; 4 regions—Hangzhou, Jiaxing, Huzhou, and Shaoxing—reported cases of H7N9 and 1 region—Ningbo—did not.

### Participants

China United Network Communications Group Co, Ltd (China Unicom) has a modern communications network characterized by nationwide coverage and global reach. China Unicom is one of China’s largest mobile phone carriers. Participation was voluntary without any financial incentive. Participants’ personal information was kept confidential and stored at China Unicom. A total of 500,000 users from the 5 examined regions in the database of China Unicom were selected. According to the registered information, mobile phone users of at least 15 years of age were sampled. Mobile phone users whose mobile phones were switched off were not studied. Participants who did not fully complete the questionnaire were excluded. In addition, after a further check of users’ information, participants whose phone number did not associate with registered information and participants who used the wireless network card phone numbers were also excluded.

### Procedure

Stratified and clustered sampling methods were employed to select the mobile phone numbers. Then a brief message was sent to the selected mobile phone users inviting them to participate in the study voluntarily. The message contained the link to an online questionnaire. The message also contained information about the investigator, the purpose of the study, and the need for a response within 24 hours. The major advantage of an Internet-based investigation is the ability to quickly recruit a large number of participants with lower cost.

### Survey Questionnaire

The online questionnaire, designed by the Zhejiang CDC, listed 16 questions on 1 page. The first 2 questions were about knowledge of H7N9: 1 addressing its symptoms and the other addressing its transmission routes. The next 4 questions were about participants’ attitudes toward H7N9. Two focused on worry and concern, and 2 on the attitude advocated by the Zhejiang CDC. Specifically, participants answered whether they worried about contracting H7N9 and the extent to which their daily lives had been influenced by H7N9. Regarding the attitude recommended by the Zhejiang CDC, participants stated whether they believed it was safe to eat cooked poultry and what they would do if they had a fever and/or a cough. Then participants answered questions about their adoption of 2 preventive practices recommended by the Zhejiang CDC, which were avoiding crowds and increasing the frequency of hand washing. Finally, participants answered sociodemographic questions about age, gender, occupation, and education. The questionnaire was executed through a mobile Internet application platform of China Unicom from April 25 to May 2, 2013. A total of 500,000 messages were sent. The study was approved by the Ethics Committee of Zhejiang Provincial CDC.

### Data Analysis

Data were analyzed using SPSS version 13.0 statistical software (SPSS, Inc, Chicago, IL, USA). Chi-square tests were used to explore if sociodemographic variables were related to KAP regarding H7N9 among mobile phone users in 5 regions of Zhejiang Province. We conducted both univariate and multivariate logistic regression analyses to investigate the associations between sociodemographic variables and KAP variables. The univariate analyses revealed the unadjusted effect of a sociodemographic variable without controlling for other sociodemographic variables. The multivariate analyses revealed the adjusted effect of a sociodemographic variable after holding other sociodemographic variables constant. Forward-selection technique was employed for model selection. We dummy-coded categorical variables. For questions about knowledge of symptoms of H7N9, knowledge of its transmission routes, attitude toward eating cooked poultry, and practice of avoiding crowds, answers of “yes” were coded into the high-KAP category indicating high levels of KAP; answers of “no” and “unclear” were coded into the low-KAP category indicating low levels of KAP. For questions about how one’s daily life had been influenced by H7N9, answers of “highly influenced” and “moderately influenced” were coded into 1 category and answers of “not influenced” were coded into the other category. For the question about recent consumption of cooked poultry, answers indicating consumption as usual were coded into 1 category and answers indicating decreased or no consumption were coded into another category. For the question about frequency of hand washing, answers indicating increased frequency were coded into the high-KAP category and answers indicating unchanged frequency or unclearness were coded into the low-KAP category. For the question about visiting a hospital with occurrence of symptoms, answers of visiting a hospital were coded into the high-KAP category whereas answers of self-treatment and no treatment were coded into the low-KAP category. A probability (*P*) value of less than .05 was considered statistically significant.

## Results

### Sociodemographic Characteristics

A total of 9582 eligible mobile phone users participated in the survey with the response rate of 1.92% (9582/500,000). With the exclusion of responses with missing data or/and logically erroneous data, 9105 of 9582 valid responses (95.02%) were included for statistical analysis. The total sample was 77.00% (7011/9105) male and 23.00% (2094/9105) female. The largest proportion (55.33%, 5038/9105) of participants were aged between 25 and 34 years. Participants involved in the poultry industry accounted for 10.94% (996/9105) of the sample. The majority (61.87%, 5633/9105) of participants had secondary education. Table 1 presents the sociodemographic characteristics of participants in the 5 regions.

**Table 1:** Cross-tabulation of sociodemographic characteristics of participants by region.

Variables		Region, n (%)					Total, n (%) (N=9105)
		Hangzhou (n=3270)	Huzhou (n=758)	Jiaxing (n=1813)	Shaoxing (n=1261)	Ningbo (n=2003)	
**Gender**							
	Male	2479 (75.81)	565 (74.54)	1443 (79.59)	972 (77.08)	1552 (77.48)	7011 (77.00)
	Female	791 (24.19)	193 (25.46)	370 (20.41)	289 (22.92)	451 (22.52)	2094 (23.00)
**Age (years)**							
	15-24	328 (10.03)	121 (15.96)	327 (18.03)	203 (16.10)	329 (16.42)	1308 (14.37)
	25-34	1901 (58.13)	379 (50.00)	1007 (55.54)	665 (52.74)	1086 (54.22)	5038 (55.33)
	35-44	740 (22.63)	164 (21.64)	343 (18.92)	263 (20.86)	408 (20.37)	1918 (21.07)
	45-54	210 (6.42)	66 (8.71)	85 (4.69)	86 (6.82)	122 (6.09)	569 (6.25)
	≥55	91 (2.78)	28 (3.69)	51 (2.81)	44 (3.48)	58 (2.90)	272 (2.99)
**Occupation**							
	Related to poultry industry	297 (9.08)	88 (11.61)	220 (12.13)	150 (11.90)	241 (12.03)	996 (10.94)
	Not related to poultry industry	2973 (90.92)	670 (88.39)	1593 (87.87)	1111 (88.10)	1762 (87.97)	8109 (89.06)
**Education**							
	Primary or less (≤6 years)	75 (2.29)	38 (5.01)	70 (3.86)	57 (4.52)	78 (3.89)	318 (3.49)
	Secondary (6-12 years)	1629 (49.82)	513 (67.68)	1333 (73.52)	823 (65.27)	1335 (66.65)	5633 (61.87)
	Postsecondary (>12 years)	1566 (47.89)	207 (27.31)	410 (22.62)	381 (30.21)	590 (29.46)	3154 (34.64)

### Knowledge Regarding H7N9

**Figure 1:**
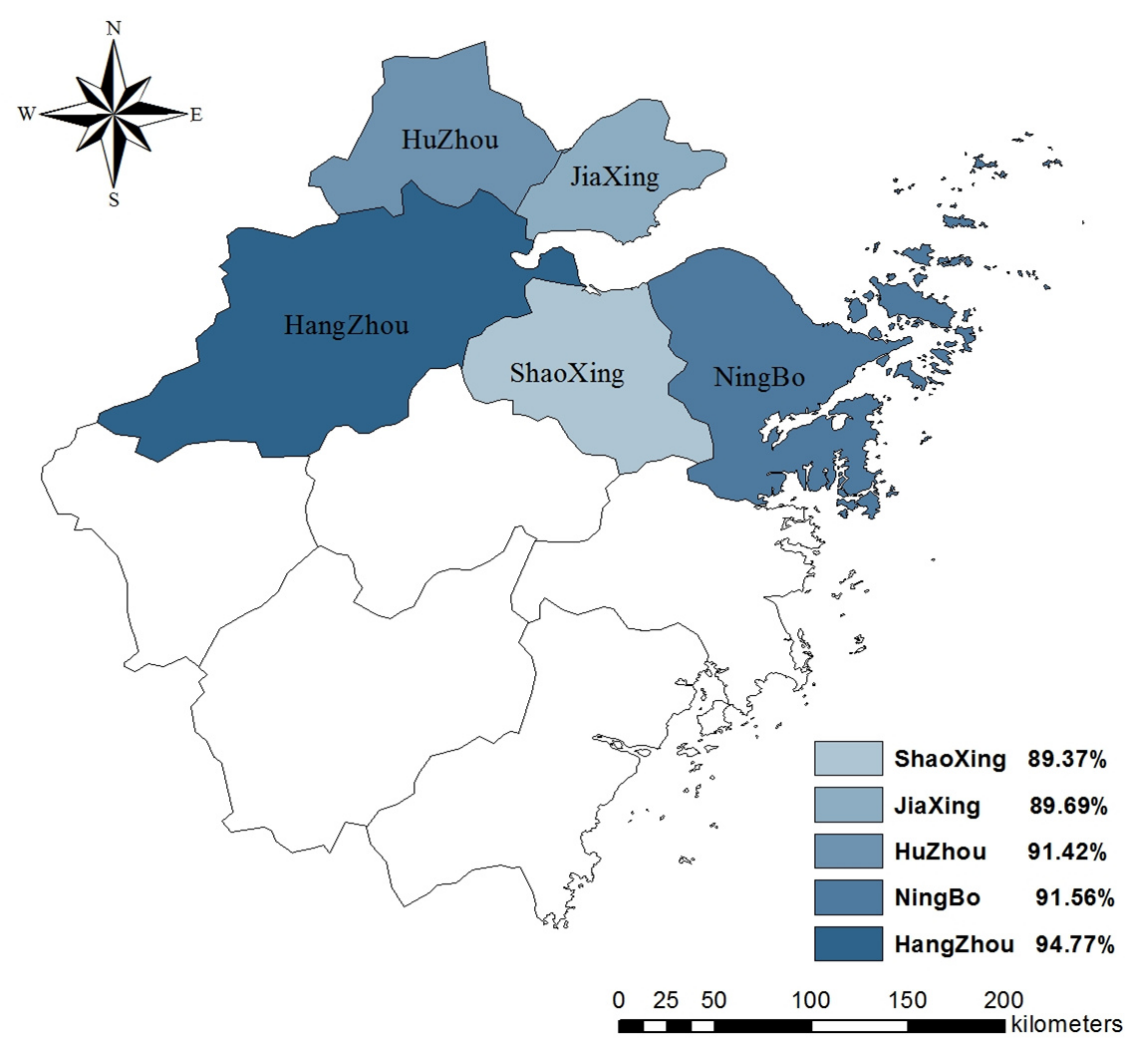
Rates of knowledge about symptoms in 5 regions.

**Figure 2:**
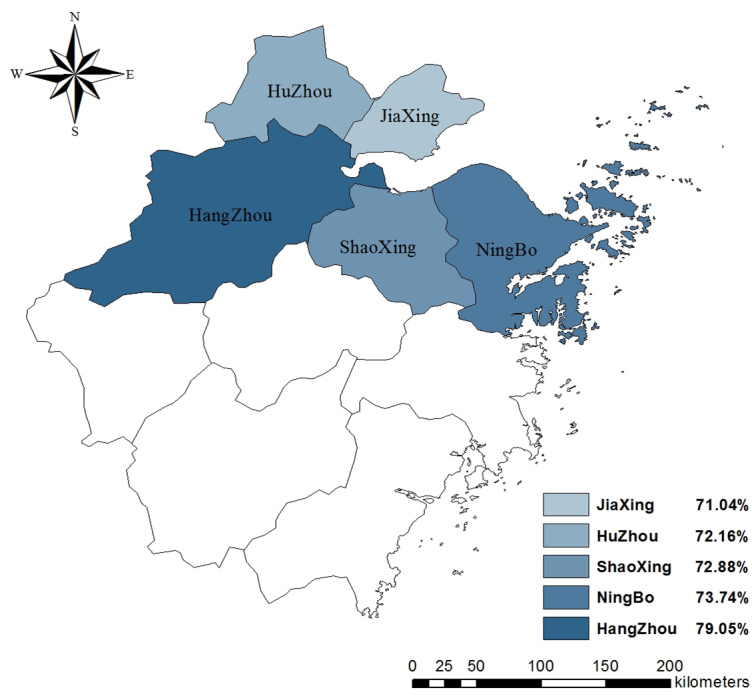
Rates of knowledge about transmission routes of H7N9 in 5 regions.

As seen in Table 2, 8379 of 9105 participants (92.03%) had some knowledge of the symptoms of H7N9 and 6816 (74.86%) had some knowledge of its transmission routes. Knowledge of H7N9 differed by region, with Hangzhou having the largest share of participants who had some knowledge about the symptoms (χ^2^_8_=63.0, *P*<.001) and transmission routes of H7N9 (χ^2^_8_=53.9, *P*<.001). The distribution of rates of knowledge about its symptoms and transmission routes in the 5 regions are displayed in Figures 1 and 2.

**Table 2:** Cross-tabulation of knowledge, attitudes, and practices regarding avian influenza A (H7N9) by region.

Variables			Region, n (%)					Total, n (%) (N=9105)	χ^2^_8_	*P*
			Hangzhou (n=3270)	Huzhou (n=758)	Jiaxing (n=1813)	Shaoxing (n=1261)	Ningbo (n=2003)			
**1. Knowledge**										
	**Symptoms of H7N9 include fever and cough**								63.0	<.001
		Yes	3099 (94.77)	693 (91.42)	1626 (89.69)	1127 (89.37)	1834 (91.56)	8379 (92.03)		
		No	40 (1.22)	11 (1.45)	38 (2.10)	23 (1.82)	38 (1.90)	150 (1.65)		
		Unclear	131 (4.01)	54 (7.12)	149 (8.22)	111 (8.80)	131 (6.54)	576 (6.33)		
	**Intimate contact with sick poultry can transmit H7N9**								53.9	<.001
		Yes	2585 (79.05)	547 (72.16)	1288 (71.04)	919 (72.88)	1477 (73.74)	6816 (74.86)		
		No	364 (11.13)	110 (14.51)	264 (14.56)	169 (13.40)	258 (12.88)	1165 (12.80)		
		Unclear	321 (9.82)	101 (13.32)	261 (14.40)	173 (13.72)	268 (13.38)	1124 (12.34)		
**2. Attitudes**										
	**It is safe to eat cooked poultry**								39.6	<.001
		Yes	2615 (79.97)	559 (73.75)	1359 (74.96)	957 (75.89)	1571 (78.43)	7061 (77.55)		
		No	285 (8.72)	83 (10.95)	238 (12.13)	151 (11.97)	204 (10.18)	961 (10.55)		
		Unclear	370 (11.31)	116 (15.30)	216 (11.91)	153 (12.13)	228 (11.38)	1083 (11.89)		
	**I am worried about contracting H7N9**								13.1	.11
		Yes	1679 (51.35)	410 (54.09)	1008 (55.60)	668 (52.97)	1049 (52.37)	4814 (52.87)		
		No	1351 (41.31)	303 (39.97)	704 (38.83)	502 (39.81)	822 (41.04)	3682 (40.44)		
		Unclear	240 (7.34)	45 (5.94)	101 (5.57)	91 (7.22)	132 (6.59)	609 (6.69)		
	**My daily life has been influenced by H7N9**								28.5	<.001
		Highly influenced	306 (9.36)	95 (12.53)	218 (12.02)	161 (12.77)	205 (10.23)	985 (10.82)		
		Moderately influenced	1696 (51.87)	401(52.90)	902(49.75)	619 (49.09)	966 (48.23)	4584 (50.35)		
		Not influenced	1268 (38.78)	262 (34.56)	693 (38.22)	481 (38.14)	832 (41.54)	3536 (38.83)		
	**If I have a fever and/or a cough, I will**								10.2	.25
		Treat myself with medication	607 (18.56)	119 (15.70)	344 (18.97)	252 (19.98)	396 (19.77)	1718 ( 18.87)		
		Visit a hospital	2585 (79.05)	619 (81.66)	1416 (78.10)	970 (76.92)	1558 (77.78)	7148 (78.51)		
		Not go for any treatment	78 (2.39)	20 (2.64)	53 (2.92)	39 (3.09)	49 (2.45)	239 (2.62)		
**3. Practices**										
	**Avoidance of crowds**								11.9	.16
		Yes	1713 (52.39)	433 (57.12)	948 (52.29)	638 (52.59)	1063 (53.07)	4795 (52.66)		
		No	1487 (45.47)	311 (41.03)	816 (45.01)	596 (47.26)	887 (44.28)	4097 (45.00)		
		Unclear	70 (2.14)	14 (1.85)	49 (2.70)	27(2.14)	53 (2.65)	213 (2.34)		
	**Frequent hand washing**								22.4	.004
		Frequency increased	1560 (47.71)	387 (51.06)	876 (48.32)	596 (47.26)	872 (43.53)	4291 (47.13)		
		Frequency unchanged	1681 (51.41)	358 (47.23)	920 (50.74)	648 (51.39)	1109 (55.37)	4716 (51.80)		
		Unclear	29 (0.89)	13 (1.72)	17(0.94)	17 (1.35)	22 (1.10)	98 (1.08)		
	**Recent consumption of cooked poultry**								37.4	<.001
		Consumption as usual	455 (13.91)	73 (9.63)	263 (14.50)	170 (13.48)	340 (16.97)	1301 (14.29)		
		Decreased consumption	1164 (35.60)	264 (34.83)	651 (35.91)	458 (36.32)	753 (37.59)	3290 (36.13)		
		No consumption	1651 (50.49)	421 (55.54)	899 (49.59)	633 (50.20)	910 (45.43)	4514 (49.58)		

### Attitudes Regarding H7N9

**Figure 3:**
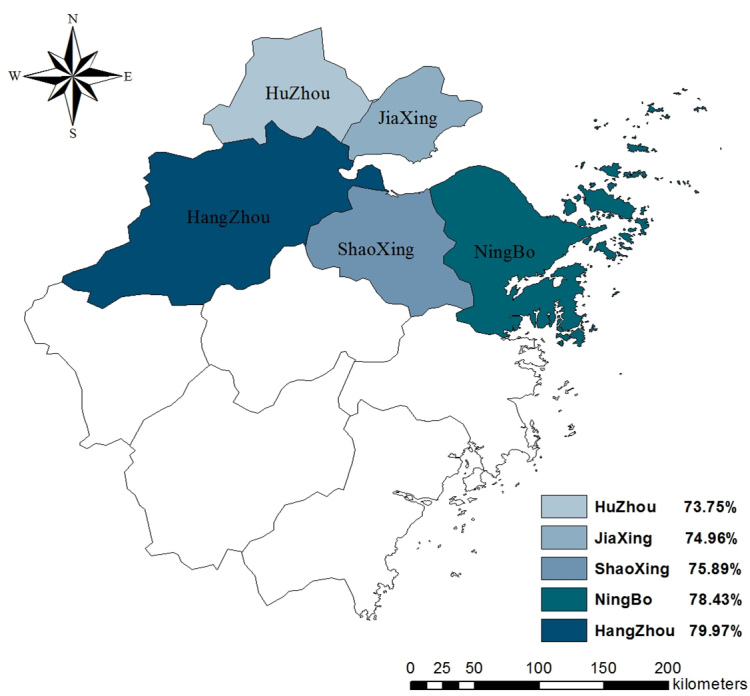
Proportion of participants who believed it was safe to eat poultry in 5 regions.

**Figure 4:**
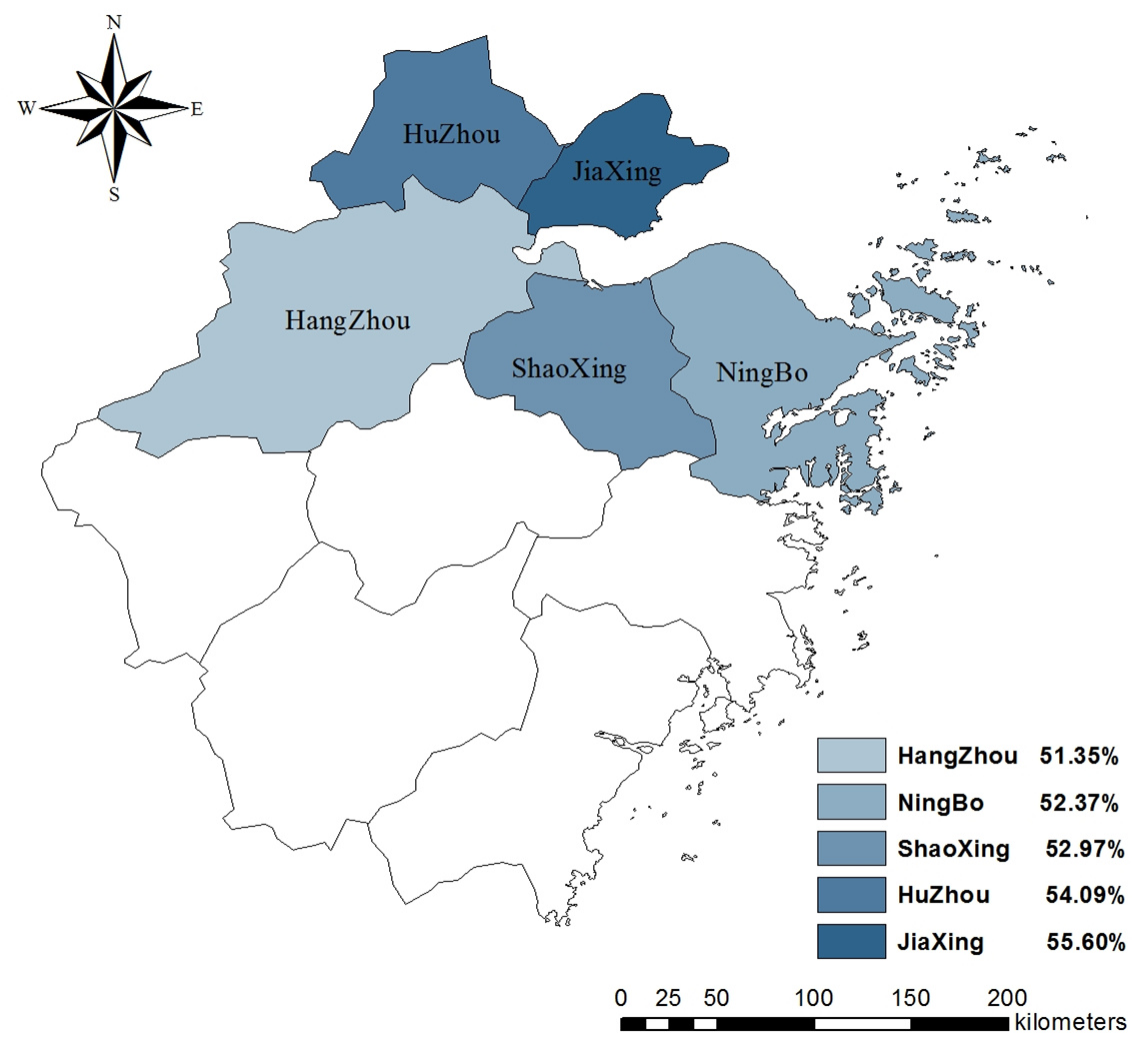
Proportion of participants who were worried about contracting H7N9 in 5 regions.

**Figure 5:**
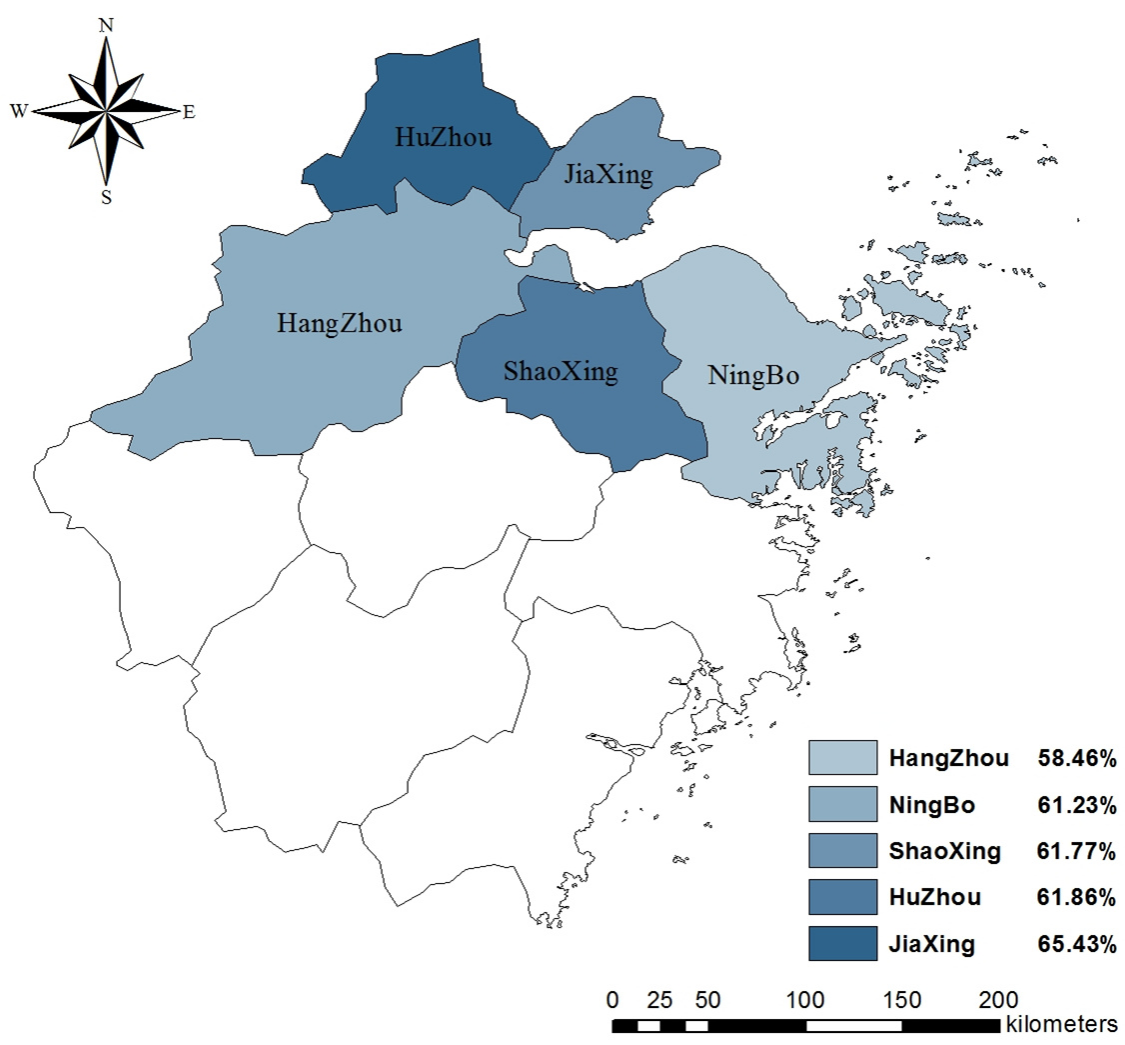
Proportion of participants who thought their daily lives had been influenced by H7N9 in 5 regions.

**Figure 6:**
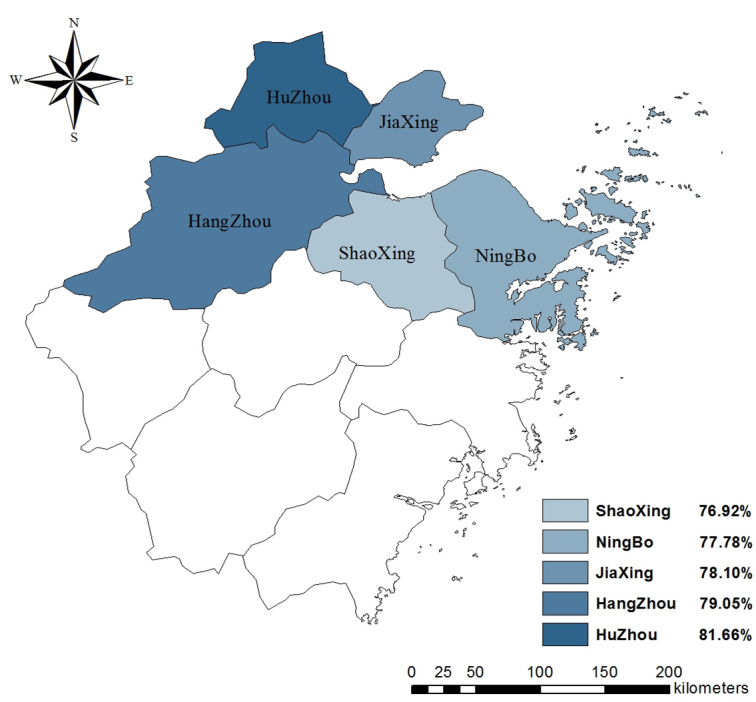
Proportion of participants who would visit a hospital if a fever and/or a cough occurred in 5 regions.

As seen in Table 2, 4814 (52.87%) participants were worried about contracting H7N9 and 8496 (61.17%) participants felt their daily lives had been affected. More than three-quarters of the participants held the attitude recommended by the Zhejiang CDC. Specifically, 7061 (77.55%) participants believed eating cooked poultry was safe and 7148 (78.51%) participants would visit a hospital if they had a fever and/or a cough. Among 4 attitude items, 2 differed by region. Attitude toward the safety of eating cooked poultry differed by region (χ^2^_8_=39.6, *P*<.001) with Hangzhou having the highest proportion of participants who thought it was safe. The attitude toward the influence of H7N9 on daily life also differed by region (χ^2^_8_=28.5, *P*<.001), with Huzhou having the highest share of participants who thought their daily lives had been affected. The distribution of rates of worrisome attitude in 5 regions are displayed in Figures 3-6.

### Practices Regarding H7N9

**Figure 7:**
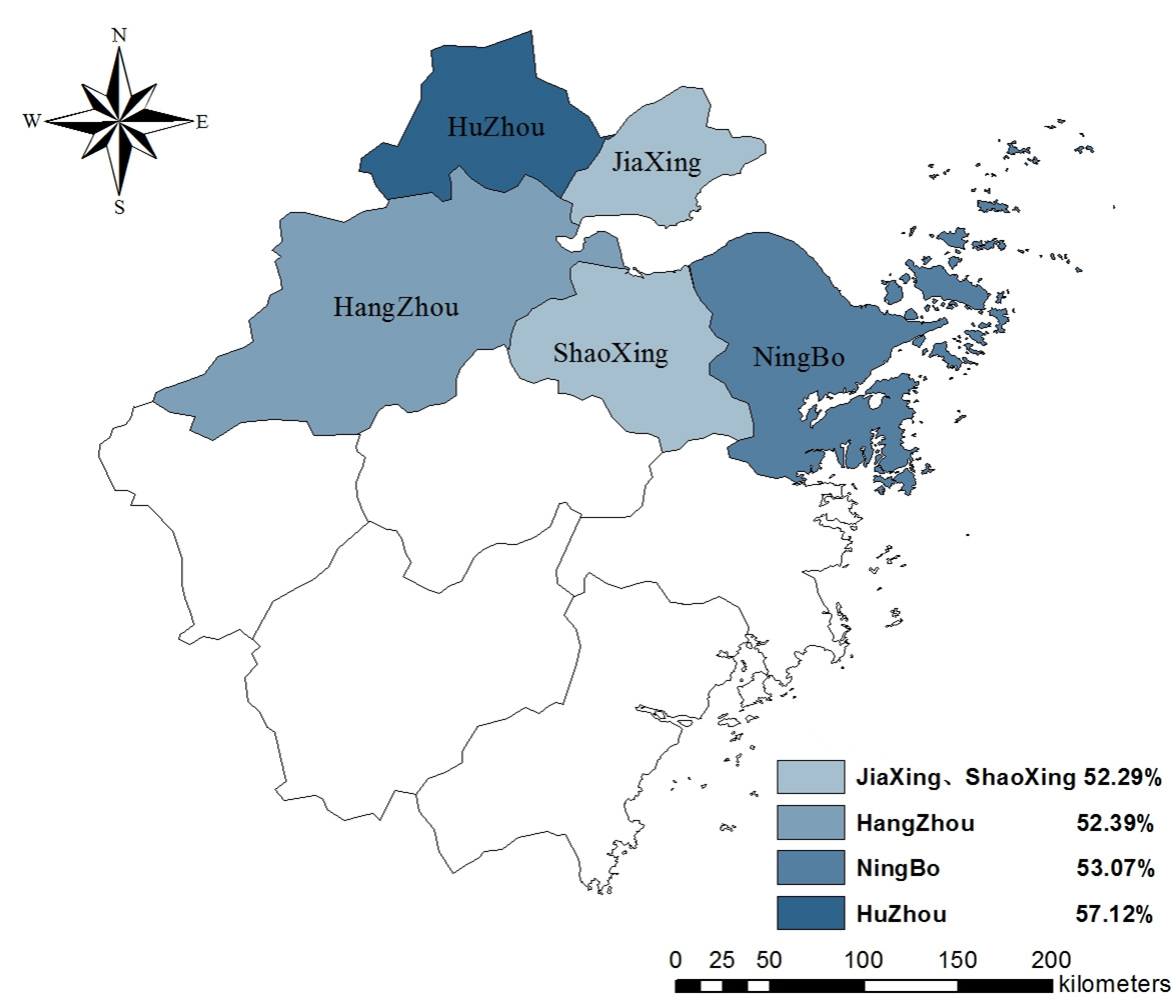
Proportion of participants who avoided crowding areas in 5 regions.

**Figure 8:**
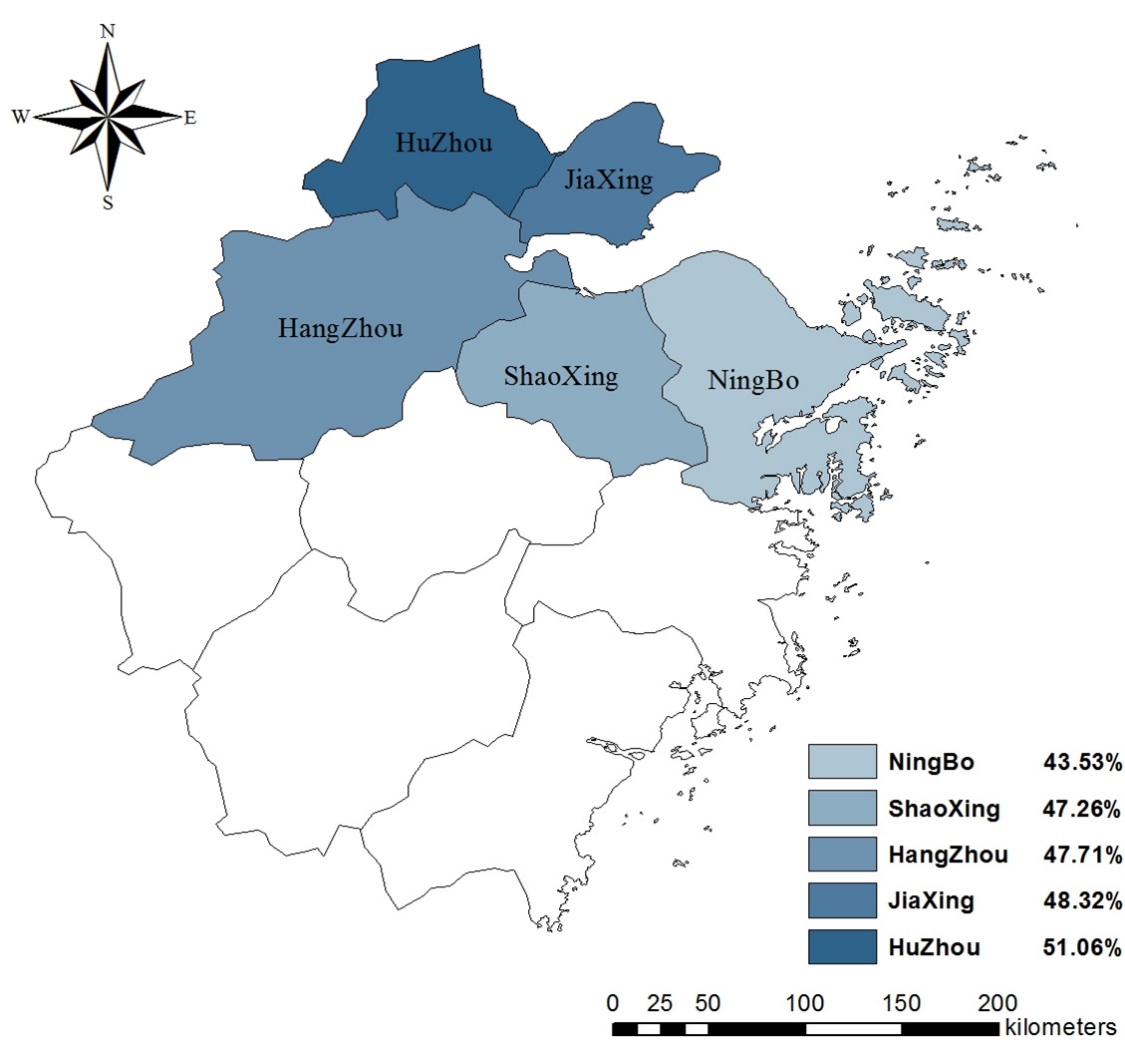
Proportion of participants who increased frequency of hand washing in 5 regions.

**Figure 9:**
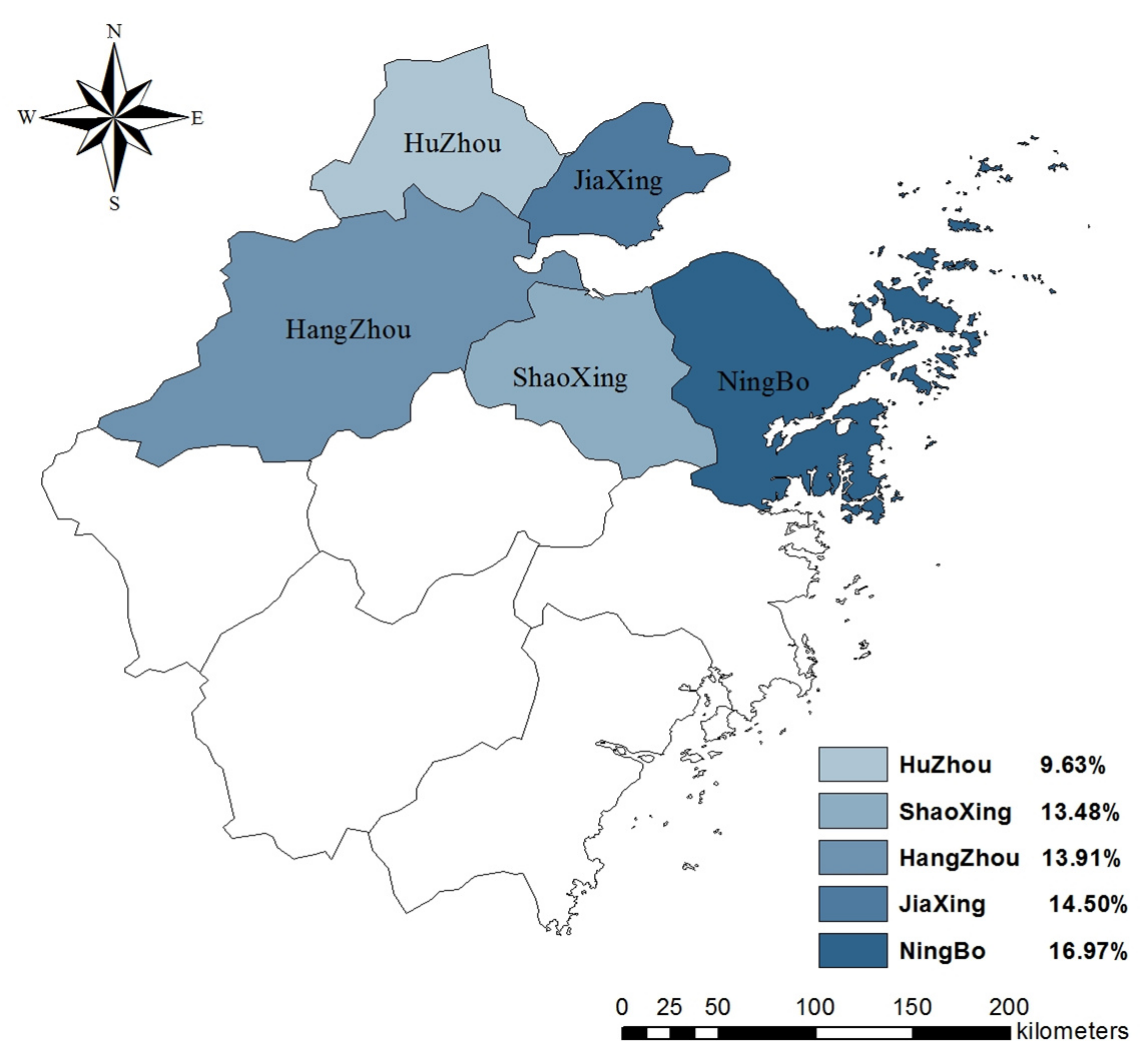
Proportion of participants decreasing or stopping eating cooked poultry in 5 regions.

As seen in Table 2, among the 3 practices recommended by the Zhejiang CDC, frequency of hand washing differed by region, with the largest share of participants from Huzhou and the smallest share of participants from Ningbo reporting increased frequency in hand washing (χ^2^_8_=22.4_,_ *P*=.004). The frequency of recent consumption of cooked poultry also differed by region (χ^2^_8_=37.4_,_ *P*<.001), with the smallest proportion of participants from Huzhou still consuming cooked poultry as usual. The distribution of rates of the 3 practices in 5 regions is displayed in Figures 7-9.

### Association Between Sociodemographic Variables and Knowledge, Attitudes, and Practices

Univariate and multivariate logistic regression analyses were conducted to investigate the association between sociodemographic variables and KAP variables. As seen in Table 3, after controlling for other sociodemographic variables, gender, age, and education were associated with knowledge about symptoms of H7N9. Age and education were associated with knowledge about transmission routes of H7N9 respectively. Female participants (OR 1.32, 95% CI 1.08-1.61) had higher odds of knowing symptoms of H7N9 than male participants. Participants aged 15-24 years had the lowest odds of knowing symptoms of H7N9 or its transmission routes as compared with other age groups. The odds of knowing symptoms of H7N9 (OR 5.05, 95% CI 3.41-7.48) and its transmission routes (OR 1.80, 95% CI 1.39-2.33) were significantly higher among participants with postsecondary or more education than those with primary or less education.

**Table 3:** Unadjusted and adjusted^a^ odds ratios (OR) and 95% confidence intervals (95% CI) of knowledge about H7N9 by sociodemographic variables.

Predictors		Knowledge about H7N9							
		Symptoms of H7N9 include fever and cough				Intimate contact with sick poultry can transmit H7N9			
		OR (95% CI)	*P*	AOR (95% CI)	*P*	OR (95% CI)	*P*	AOR (95% CI)	*P*
**Gender**									
	Male (reference)	1		1		1		NA	
	Female	1.40 (1.15, 1.71)	.001	1.32 (1.08, 1.61)	.01	1.03 (0.92, 1.15)	.67		
**Age (years)**									
	15-24 (reference)	1		1		1		1	
	25-34	1.54 (1.28, 1.87)	<.001	1.26 (1.04, 1.53)	.02	1.55 (1.36, 1.76)	<.001	1.38 (1.21, 1.58)	<.001
	35-44	2.61 (2.01, 3.38)	<.001	2.22 (1.71, 2.88)	<.001	2.08 (1.77, 2.44)	<.001	1.89 (1.61, 2.22)	<.001
	45-54	2.77 (1.83, 4.19)	<.001	2.77 (1.83, 4.21)	<.001	2.30 (1.81, 2.92)	<.001	2.25 (1.77, 2.86)	<.001
	≥55	6.36 (2.78, 4.51)	<.001	7.82 (3.38, 18.06)	<.001	1.68 (1.24, 2.27)	.001	1.72 (1.26, 2.34)	.001
**Occupation**									
	Related to poultry industry (reference)	1		NA		1		NA	
	Not related to poultry industry	1.20 (0.95, 1.51)	.12			1.18 (1.02, 1.37)	.03		
**Education**									
	Primary or less (≤6 years) (reference)	1		1		1		1	
	Secondary (6-12 years)	1.32 (0.94, 1.85)	.11	1.78 (1.25, 2.54)	.001	1.04 (0.81, 1.33)	.78	1.04 (0.81, 1.33)	.78
	Postsecondary (>12 years)	3.87 (2.66, 5.65)	<.001	5.05 (3.41, 7.48)	<.001	1.80 (1.39, 2.44)	<.001	1.80 (1.39, 2.33)	<.001

^a^ Multivariate logistic regression, adjusting for the other factors shown in the table; AOR=adjusted odds ratio.

Table 4 shows the associations between sociodemographic variables and attitude variables. After controlling for other sociodemographic variables, gender was associated with 3 of 4 attitude items. Female participants were more likely to worry about H7N9 infection (OR 1.15, 95% CI 1.04-1.27), to feel that their daily lives had been affected (OR 1.27, 95% CI 1.15-1.41), and to go to a hospital if a fever and/or a cough occurred (OR 1.25, 95% CI 1.01-1.41). Male and female participants did not differ in their attitude toward safety of eating cooked poultry though. The attitudinal effect of age varied by attitude item; for example, participants aged 15-24 years had the lowest odds of believing that it was safe to eat cooked poultry as compared with other age groups. They did not differ from those aged 25-34 years or those aged 55 years and older in the odds of worrying about H7N9 infection. In comparison, participants aged 45-54 years and 35-44 years had lower odds of worrying about H7N9. Participants aged 15-24 years were not different from those aged 25-34 years in the odds of visiting a hospital at the occurrence of a fever and/or a cough. Participants aged 35-44 years (OR 1.69, 95% CI 1.42-2.01), 45-54 years (OR 1.60, 95% CI 1.24-2.06), and 55 years and older (OR 1.91, 95% CI 1.34-2.73) were more likely to visit a hospital than participants aged 15-24 years. Compared to participants whose occupation was related to the poultry industry, others had lower odds of worrying about H7N9 infection (OR 0.78, 95% CI 0.68-0.90) and to feel that their daily life had been affected (OR 0.45, 95% CI 0.38-0.52). Odds of believing in the safety of eating cooked poultry increased by education level. Compared to participants with primary or less education, participants with postsecondary or more education had approximately 3 times the odds (OR 2.81, 95% CI 2.18-3.63) of believing that eating cooked poultry was safe. Meanwhile, they were less likely to worry about H7N9 infection (OR 0.69, 95% CI 0.54-0.88).

**Table 4:** Unadjusted and adjusted^a^ odds ratios (OR) and 95% confidence intervals (95% CI) of attitude toward H7N9 by sociodemographic variables.

Predictors		Attitude about H7N9															
		It is safe to eat cooked poultry				I am worried about contracting H7N9				My daily life has been influenced by H7N9				If I have a fever and/or cough, I will visit a hospital			
		OR (95% CI)	*P*	AOR (95% CI)	*P*	OR (95% CI)	*P*	AOR (95% CI)	*P*	OR (95% CI)	*P*	AOR (95% CI)	*P*	OR (95% CI)	*P*	AOR (95% CI)	*P*
**Gender**																	
	Male (reference)	1				1		1		1		1		1		1	
	Female	0.96 (0.85, 1.08)	.48			1.11 (1.00, 1.22)	.047	1.15 (1.04, 1.27)	.007	1.22 (1.10, 1.35)	<.001	1.27 (1.15, 1.41)	<.001	1.22 (1.08, 1.38)	.002	1.25 (1.10, 1.41)	<.001
**Age (years)**																	
	15-24 (reference)	1		1		1		1		1		1		1		1	
	25-34	1.38 (1.21, 1.58)	<.001	1.17 (1.02, 1.35)	.03	0.94 (0.83, 1.07)	.35	1.01 (0.90, 1.15)	.82	1.07 (0.95, 1.21)	.27	1.11 (0.98, 1.25)	.12	1.08 (0.94, 1.25)	.27	1.08 (0.94, 1.24)	.29
	35-44	1.89 (1.60, 2.23)	<.001	1.65 (1.39, 1.95)	<.001	0.81 (0.70, 0.93)	.004	0.86 (0.75, 1.00)	.04	1.14 (0.98, 1.31)	.08	1.15 (1.00, 1.33)	.06	1.68 (1.41, 2.00)	<.001	1.69 (1.42, 2.01)	<.001
	45-54	2.43 (1.87, 3.15)	<.001	2.40 (1.84, 3.12)	<.001	0.64 (0.53, 0.79)	<.001	0.65 (0.53, 0.79)	<.001	1.01 (0.83, 1.24)	.90	0.98 (0.80, 1.20)	.84	1.59 (1.24, 2.04)	<.001	1.60 (1.24, 2.06)	<.001
	≥55	1.91 (1.37, 2.66)	<.001	2.14 (1.52, 3.02)	<.001	1.05 (0.81, 1.37)	.72	0.93 (0.70, 1.22)	.59	2.12 (1.57, 2.85)	<.001	1.49 (1.09, 2.03)	.01	1.86 (1.31, 2.67)	.001	1.91 (1.34, 2.73)	<.001
**Occupation**																	
	Related to poultry industry (reference)	1				1		1		1		1		1			
	Not related to poultry industry	1.14 (0.98, 1.33)	.10			0.76 (0.66, 0.87)	<.001	0.78 (0.68, 0.90)	<.001	0.44 (0.38, 0.52)	<.001	0.45 (0.38, 0.52)	<.001	0.86 (0.73, 1.01)	.07		
**Education**																	
	Primary or less (≤6 years) (reference)	1		1		1		1		1							
	Secondary (6-12 years)	1.29 (1.01, 1.64)	.04	1.53 (1.19, 1.98)	.001	0.89 (0.70, 1.11)	.30	0.91 (0.72, 1.16)	.46	0.64 (0.50, 0.82)	<.001			1.15 (0.88, 1.49)	.31		
	Postsecondary (>12 years)	2.81 (2.18, 3.63)	<.001	2.81 (2.18, 3.63)	<.001	0.67 (0.53, 0.85)	.001	0.69 (0.54, 0.88)	.003	0.64 (0.50, 0.83)	.001			1.22 (0.93, 1.60)	.14		

^a^ Multivariate logistic regression, adjusting for the other factors shown in the table; AOR=adjusted odds ratio.

Table 5 shows the associations between sociodemographic variables and practice variables. After controlling for other sociodemographic variables, gender was associated with the 3 preventive practices recommended by the Zhejiang CDC. Female participants had higher odds to implement the practices than males (avoidance of crowds: OR 1.38, 95% CI 1.25-1.52; frequent hand washing: OR 1.24, 95% CI 1.12-1.36; decreasing consumption of cooked poultry: OR 2.19, 95% CI 1.85-2.60). The odds of using the advocated practices generally increased by age, with participants aged 55 years and older more likely and participants aged 15-24 years least likely to adopt advocated practices. Occupation was only associated with the frequency of hand washing, with participants not involved in the poultry industry being less likely to increase hand washing (OR 0.65, 95% CI 0.56-0.75).

**Table 5:** Unadjusted and adjusted^a^ odds ratios (OR) and 95% confidence intervals (95% CI) of practices regarding H7N9 by sociodemographic variables.

Predictors		Practices about H7N9											
		Avoidance of crowds				Frequent hand washing				Decreased or no consumption of cooked poultry			
		OR (95% CI)	*P*	AOR (95% CI)	*P*	OR (95% CI)	*P*	AOR (95% CI)	*P*	OR (95% CI)	*P*	AOR (95% CI)	*P*
**Gender**													
	Male (reference)	1		1		1		1		1		1	
	Female	1.34 (1.22, 1.48)	<.001	1.38 (1.25, 1.52)	<.001	1.18 (1.07, 1.30)	.001	1.24 (1.12, 1.36)	<.001	2.18 (1.84, 2.58)	<.001	2.19 (1.85, 2.60)	<.001
**Age (years)**													
	15-24 (reference)	1		1		1		1		1		1	
	25-34	1.12 (1.00, 1.27)	.06	1.23 (1.00, 1.27)	.06	0.99 (0.88, 1.12)	.87	1.02 (0.90, 1.15)	.77	1.33 (1.13, 1.56)	.001	1.30 (1.11, 1.53)	.001
	35-44	1.43 (1.24, 1.65)	<.001	1.44 (1.25, 1.66)	<.001	1.03 (0.90, 1.19)	.64	1.06 (0.92, 1.22)	.44	1.73 (1.42, 2.10)	<.001	1.74 (1.43, 2.12)	<.001
	45-54	1.70 (1.39, 2.01)	<.001	1.70 (1.39, 2.08)	<.001	1.31 (1.07, 1.59)	.01	1.31 (1.07, 1.59)	.01	1.87 (1.39, 2.51)	<.001	1.94 (1.44, 2.62)	<.001
	≥55	1.76 (1.34, 2.29)	<.001	1.67 (1.27, 2.20)	<.001	1.79 (1.37, 2.34)	<.001	1.57 (1.19, 2.08)	.002	1.71 (1.15, 2.54)	.01	2.06 (1.37, 3.10)	.001
**Occupation**													
	Related to poultry industry (reference)	1		1		1		1		1		1	
	Not related to poultry industry	0.81 (0.71, 0.92)	.001	0.84 (0.73, 0.97)	.02	0.62 (0.55, 0.71)	<.001	0.65 (0.56, 0.75)	<.001	1.22 (1.02, 1.46)	.03	1.24 (1.03, 1.49)	.03
**Education**													
	Primary or less (≤6 years) (reference)	1		NA		1		NA		1		NA	
	Secondary (6-12 years)	0.89 (0.71, 1.11)	.30			0.99 (0.79, 1.24)	.91			1.18 (0.87, 1.61)	.28		
	Postsecondary (>12 years)	0.91 (0.72, 1.14)	.41			0.88 (0.70, 1.11)	.28			1.18 (0.86, 1.61)	.31		

^a^ Multivariate logistic regression, adjusting for the other factors shown in the table; AOR=adjusted odds ratio.

## Discussion

### Principal Results

Control and prevention of a new infectious disease need public participation. In order to respond appropriately to the outbreak of an infectious disease, such as H7N9, people need to have basic knowledge about its symptoms and transmission routes. Our study showed that mobile Internet users in Zhejiang Province knew more about the symptoms of H7N9 than about its transmission routes. This showed that people lacked knowledge of how this new communicable disease was transmitted. The finding suggests that in the outbreak of a new infectious disease, the government as well as medical institutions should carry out public health education not only about its symptoms but also about its transmission routes.

In Zhejiang Province, there has been a great demand for live poultry, such as live chickens and ducks, among the general population. After the emergence of H7N9, the provincial government took preventive strategies, such as closing live poultry markets and conducting public health education. Meanwhile, more than half of the participants indicated that they worried about contracting H7N9 and that their daily lives had been influenced by H7N9. This may be derived from the increasing amount of media coverage of the epidemic and mortality, lack of knowledge about its transmission routes, and closings of live poultry markets.

The literature has shown that hand hygiene can reduce the spread of respiratory disease efficiently [13]. Our study revealed that approximately half of the participants washed their hands more frequently. The tendency to adopt precautionary behaviors to prevent disease infection was seen in previous studies of other types of influenza viruses, such as H1N1 [14-16].

Knowledge of H7N9 differed by region, with Hangzhou having the largest share of participants who knew the symptoms and transmission routes of H7N9. In terms of attitude, Hangzhou also saw the highest proportion of participants who believed it was safe to eat cooked poultry. This may be related to the fact that Hangzhou saw the greatest number of H7N9 cases [2].

In comparison, worry about contracting H7N9 did not differ by region. There was also no difference in region regarding avoidance of crowds and frequency of hand washing. The consumption of cooked poultry differed by region, with Huzhou seeing the largest share of participants decreasing or stopping eating cooked poultry.

Previous studies revealed that demographic factors, such as age, and gender, increased pandemic knowledge, and higher risk perception induced people to adopt preventive practices [17]. Our data indicated that sociodemographic variables were significantly associated with KAP. For example, female participants were more likely to know about symptoms of H7N9, to worry about it, and to report their lives being influenced by it. They were also more likely to take the recommended precautionary practices, such as avoiding crowds and washing hands more frequently, to prevent H7N9 infection. The findings were consistent with the literature, which indicated greater likelihood for females than males to perceive higher levels of risk of contracting contagious diseases [18,19], and to take precautionary measures to prevent getting such diseases [20]. The findings implied a plausible relationship between knowledge and the adoption of preventive practices, as suggested in the literature [21,22], among females. Therefore, enhancing knowledge about a new infectious disease can be an effective strategy to encourage the adoption of preventive measures. The findings also implied a plausible association between risk perception and the adoption of preventive practices among females. This may also apply to participants with occupations related to poultry industry. Specifically, participants with occupations related to the poultry industry were more likely to worry about contracting H7N9 and to report their lives being influenced by H7N9. They were also more likely to take precautionary practices. These results suggest the behavioral effect of risk perception. However, increased worry and concern did not seem to elicit behavioral change among young people or among participants with less education. Young participants aged 15-24 years were more likely to worry about contracting H7N9, but were less likely to comply with advocated protective measures. Education affected attitude toward H7N9; participants with primary education or lower tended to worry about contracting H7N9 and feel that their lives were being affected. But education did not alter the likelihood of taking preventive practices.

In sum, male participants and younger participants were less likely to comply with advocated protective practices, which may imply high risks of contracting H7N9. The difference was that male participants worried less, but young participants worried more about contracting H7N9. The findings suggest the need for more effective materials to promote advocated preventive practices for the high-risk groups and young male individuals, in particular. In addition, the government also needs to develop strategies to encourage the public to change the habit of killing live poultry or purchasing chilled poultry products. To summarize, the content and the form of health education should be designed in accordance with the sociodemographics of the target population to increase accessibility and efficiency.

### Limitations

This study has some limitations. First, the study is limited by the volunteer sample of mobile Internet users from 5 regions of Zhejiang Province. Those who cared more about public health may have participated in the study. Thus, the results may not be generalizable to other residents who were not mobile Internet users. Second, the possibility of social desirability bias as a result of the self-reported survey design may exist, which may have led to bias in reported KAP levels. Finally, this was an exploratory study designed as a quick method to understand levels of KAP about H7N9 among mobile Internet users. Considering people may not want to respond to a long questionnaire, we kept the questionnaire short. This may have increased the response rate, but limited the depth of the questions.

### Conclusions

Our study provides valuable insights into KAP related to H7N9 among mobile Internet users in 5 regions of Zhejiang Province. There were some regional differences in KAP. Hangzhou, the region that saw the greatest number of H7N9 infections, saw the largest proportion of participants with basic knowledge of H7N9. The associations between sociodemographic variables and KAP suggest that government agencies and medical institutions need to enhance public health education for potentially high-risk groups, such as males, younger people, and people with occupations related to the poultry industry, in order to encourage them to take preventive behaviors. Meanwhile, more education about the disease and, in particular, its transmission routes should be conducted among those with less education.

## Abbreviations

AOR: adjusted odds ratio
H7N9: avian influenza A
KAP: knowledge, attitudes, and practices
SARS: severe acute respiratory syndrome

## References

[1] World Health Organization.

[2] Gong Z, Lv H, Ding H, Han J, Sun J, Chai C, Cai J, Yu Z, Chen E (2014). Epidemiology of the avian influenza A (H7N9) outbreak in Zhejiang Province, China. BMC Infect Dis.

[3] Li Q, Zhou L, Zhou M, Chen Z, Li F, Wu H, Xiang N, Chen E, Tang F, Wang D, Meng L, Hong Z, Tu W, Cao Y, Li L, Ding F, Liu B, Wang M, Xie R, Gao R, Li X, Bai T, Zou S, He J, Hu J, Xu Y, Chai C, Wang S, Gao Y, Jin L, Zhang Y, Luo H, Yu H, He J, Li Q, Wang X, Gao L, Pang X, Liu G, Yan Y, Yuan H, Shu Y, Yang W, Wang Y, Wu F, Uyeki TM, Feng Z (2014). Epidemiology of human infections with avian influenza A(H7N9) virus in China. N Engl J Med.

[4] Wu S, Wu F, He J (2013). Emerging risk of H7N9 influenza in China. Lancet.

[5] Timothy M, Nancy J, Uyeki (2013). Cox. Global Concerns Regarding Novel Influenza A (H7N9) Virus Infections. N Engl J Med.

[6] Blendon RJ, Benson JM, DesRoches CM, Raleigh E, Taylor-Clark K (2004). The public's response to severe acute respiratory syndrome in Toronto and the United States. Clin Infect Dis.

[7] Seale H, Heywood AE, McLaws ML, Ward KF, Lowbridge CP, Van D, MacIntyre CR (2010). Why do I need it? I am not at risk! Public perceptions towards the pandemic (H1N1) 2009 vaccine. BMC Infect Dis.

[8] Goodwin R, Haque S, Neto F, Myers LB (2009). Initial psychological responses to Influenza A, H1N1 ("Swine flu"). BMC Infect Dis.

[9] Horby P (2013). H7N9 is a virus worth worrying about. Nature.

[10] China Internet Network Information Center Beijing: China Internet Network Information Center.

[11] Zhejiang Communication Management Bureau.

[12] Eysenbach G (2004). Improving the quality of Web surveys: the Checklist for Reporting Results of Internet E-Surveys (CHERRIES). J Med Internet Res.

[13] NSW Department of Health.

[14] Lau JT, Griffiths S, Choi KC, Tsui HY (2009). Widespread public misconception in the early phase of the H1N1 influenza epidemic. J Infect.

[15] Akan H, Gurol Y, Izbirak G, Ozdatli S, Yilmaz G, Vitrinel A, Hayran O (2010). Knowledge and attitudes of university students toward pandemic influenza: a cross-sectional study from Turkey. BMC Public Health.

[16] Xiang N, Shi Y, Wu J, Zhang S, Ye M, Peng Z, Zhou L, Zhou H, Liao Q, Huai Y, Li L, Yu Z, Cheng X, Su W, Wu X, Ma H, Lu J, McFarland J, Yu H (2010). Knowledge, attitudes and practices (KAP) relating to avian influenza in urban and rural areas of China. BMC Infect Dis.

[17] Askarian M, Danaei M, Vakili V (2013). Knowledge, Attitudes, and Practices Regarding Pandemic H1N1 Influenza Among Medical and Dental Residents and Fellowships in Shiraz, Iran. Int J Prev Med.

[18] Leung GM, Ho LM, Chan SK, Ho SY, Bacon-Shone J, Choy RY, Hedley AJ, Lam TH, Fielding R (2005). Longitudinal assessment of community psychobehavioral responses during and after the 2003 outbreak of severe acute respiratory syndrome in Hong Kong. Clin Infect Dis.

[19] Tooher R, Collins JE, Street JM, Braunack-Mayer A, Marshall H (2013). Community knowledge, behaviours and attitudes about the 2009 H1N1 Influenza pandemic: a systematic review. Influenza Other Respir Viruses.

[20] Yap J, Lee VJ, Yau TY, Ng TP, Tor PC (2010). Knowledge, attitudes and practices towards pandemic influenza among cases, close contacts, and healthcare workers in tropical Singapore: a cross-sectional survey. BMC Public Health.

[21] Lin Y, Huang L, Nie S, Liu Z, Yu H, Yan W, Xu Y (2011). Knowledge, attitudes and practices (KAP) related to the pandemic (H1N1) 2009 among Chinese general population: a telephone survey. BMC Infect Dis.

[22] Brooks-Pollock E, Tilston N, Edmunds WJ, Eames KT (2011). Using an online survey of healthcare-seeking behaviour to estimate the magnitude and severity of the 2009 H1N1v influenza epidemic in England. BMC Infect Dis.

